# Supplementary figures and images for: TMEM98 is a negative regulator of FRAT mediated Wnt/ß-catenin signalling
Source: PLoS One. 2020 Jan 21;15(1):e0227435. doi: 10.1371/journal.pone.0227435 (PMC6974163; doi:10.1371/journal.pone.0227435)

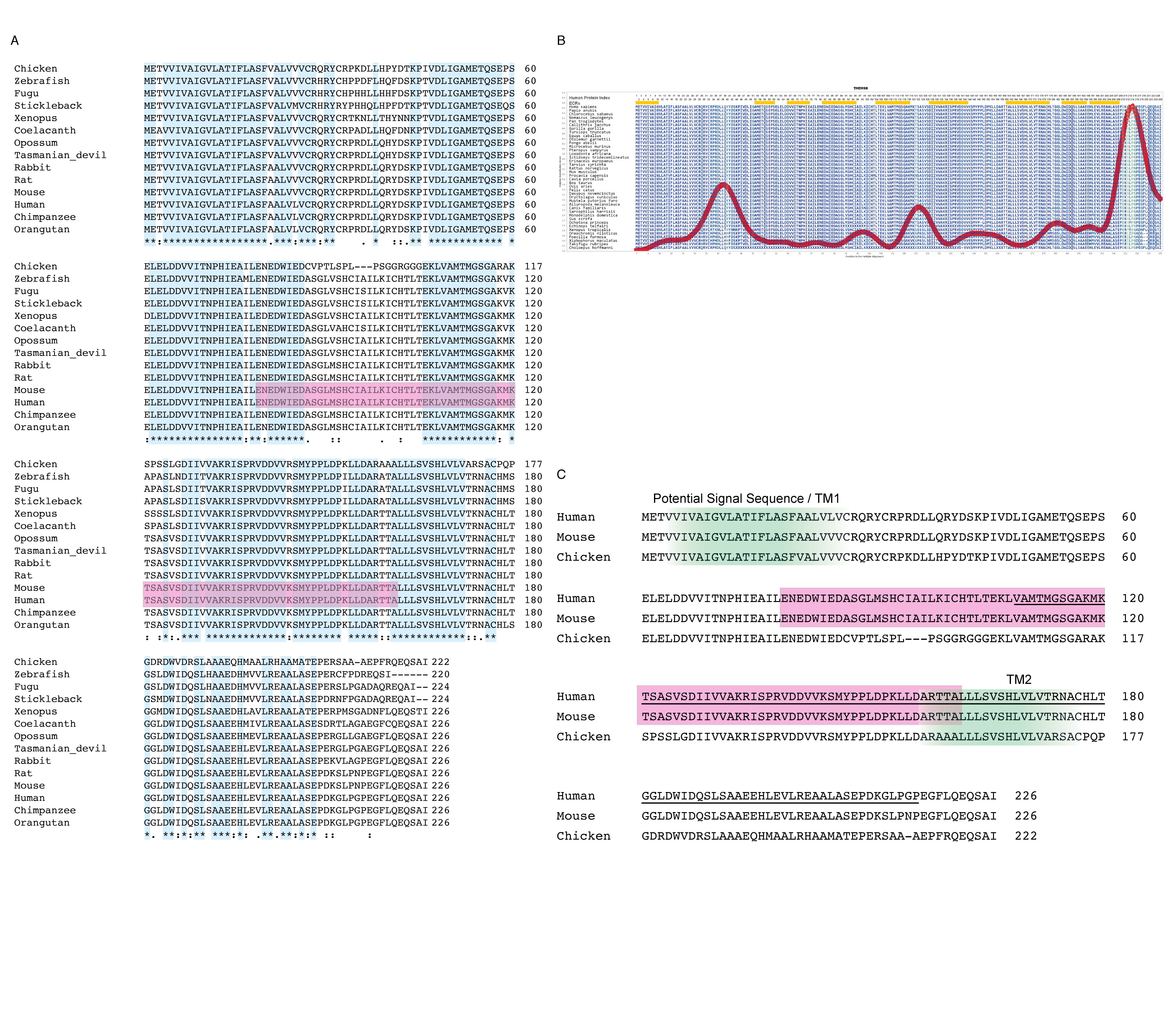

Supplement: S1 Fig — (TIF) [file pone.0227435.s001.tif]

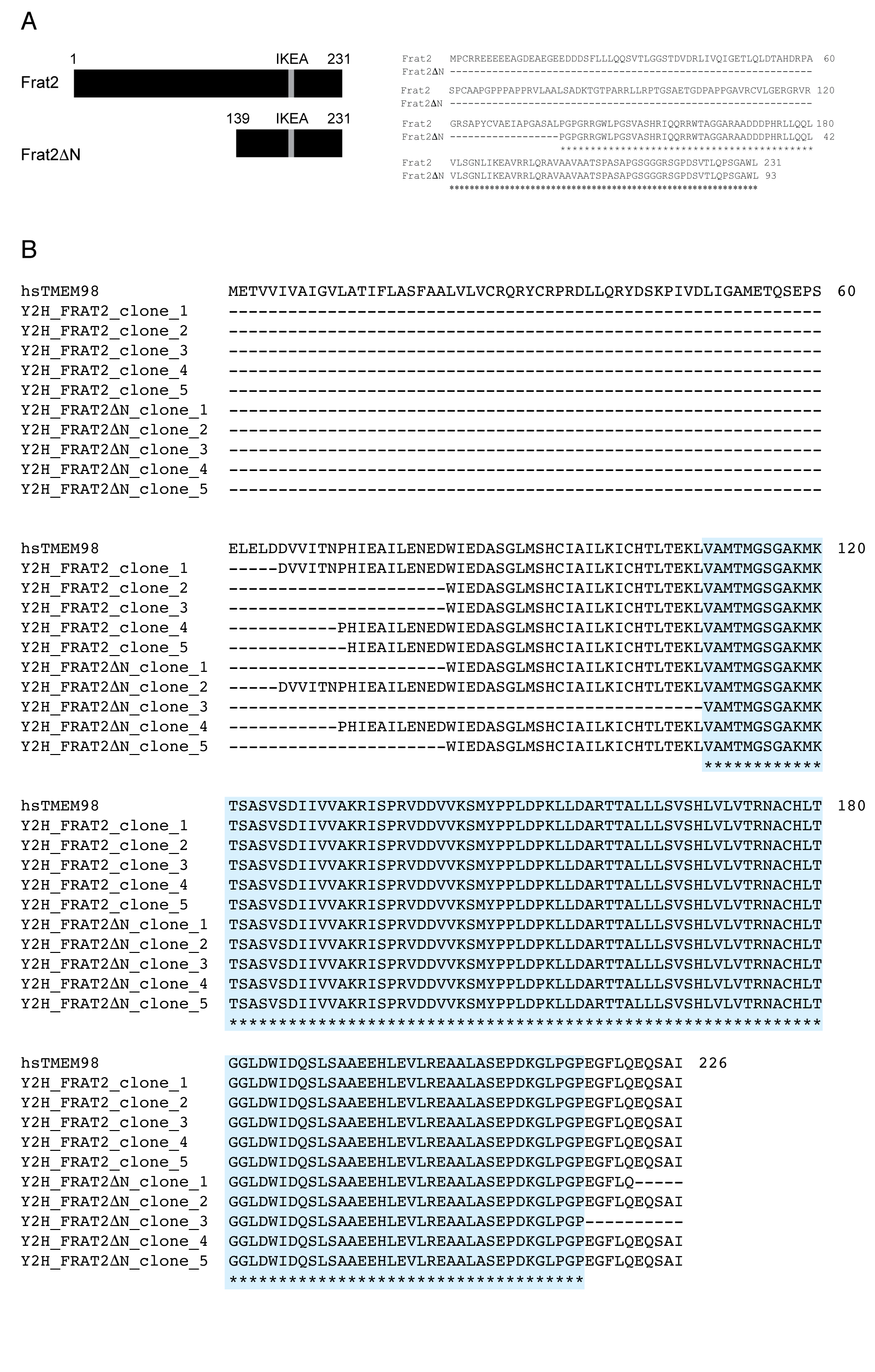

Supplement: S2 Fig — (TIF) [file pone.0227435.s002.tif]

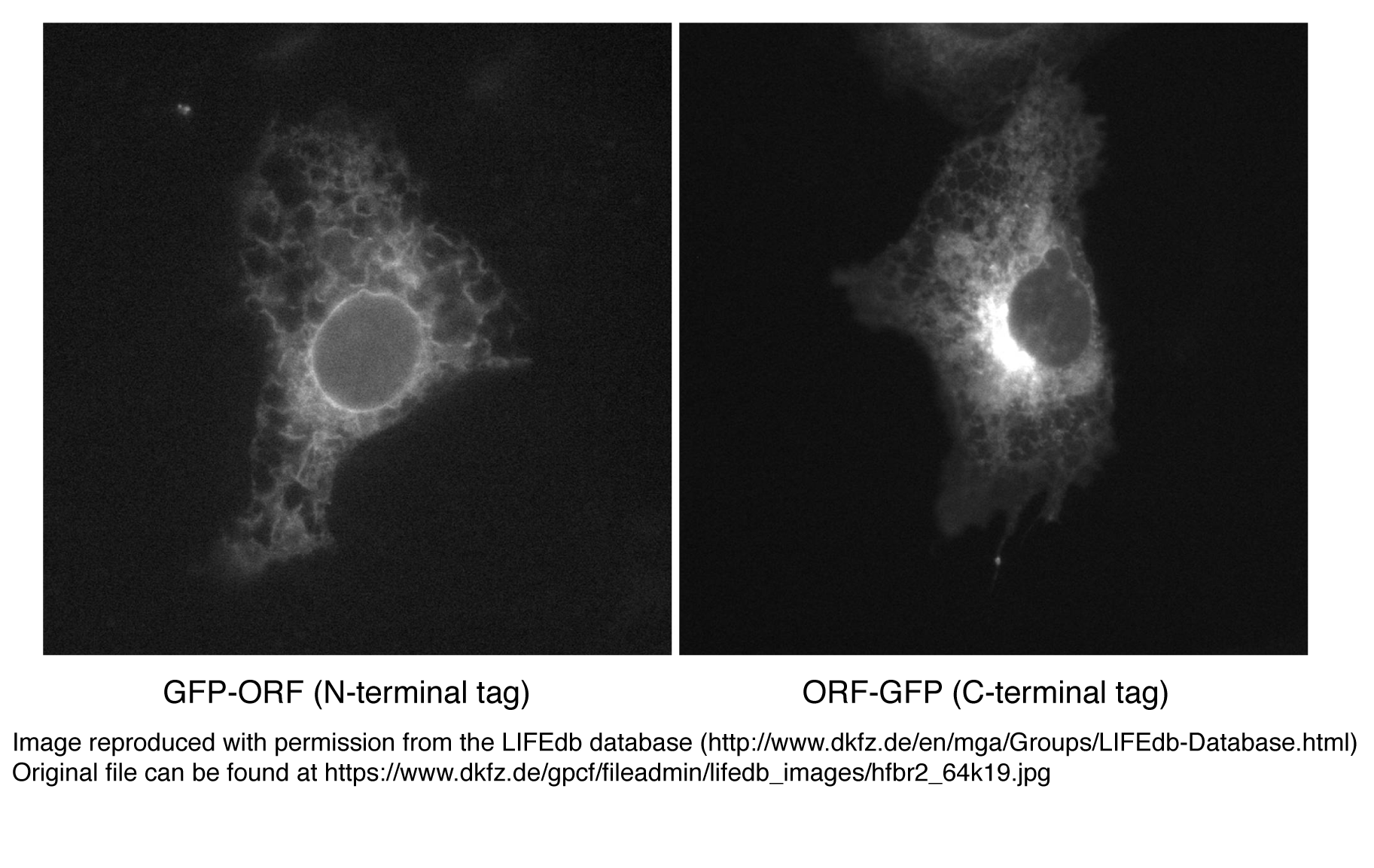

Supplement: S3 Fig — (TIF) [file pone.0227435.s003.tif]

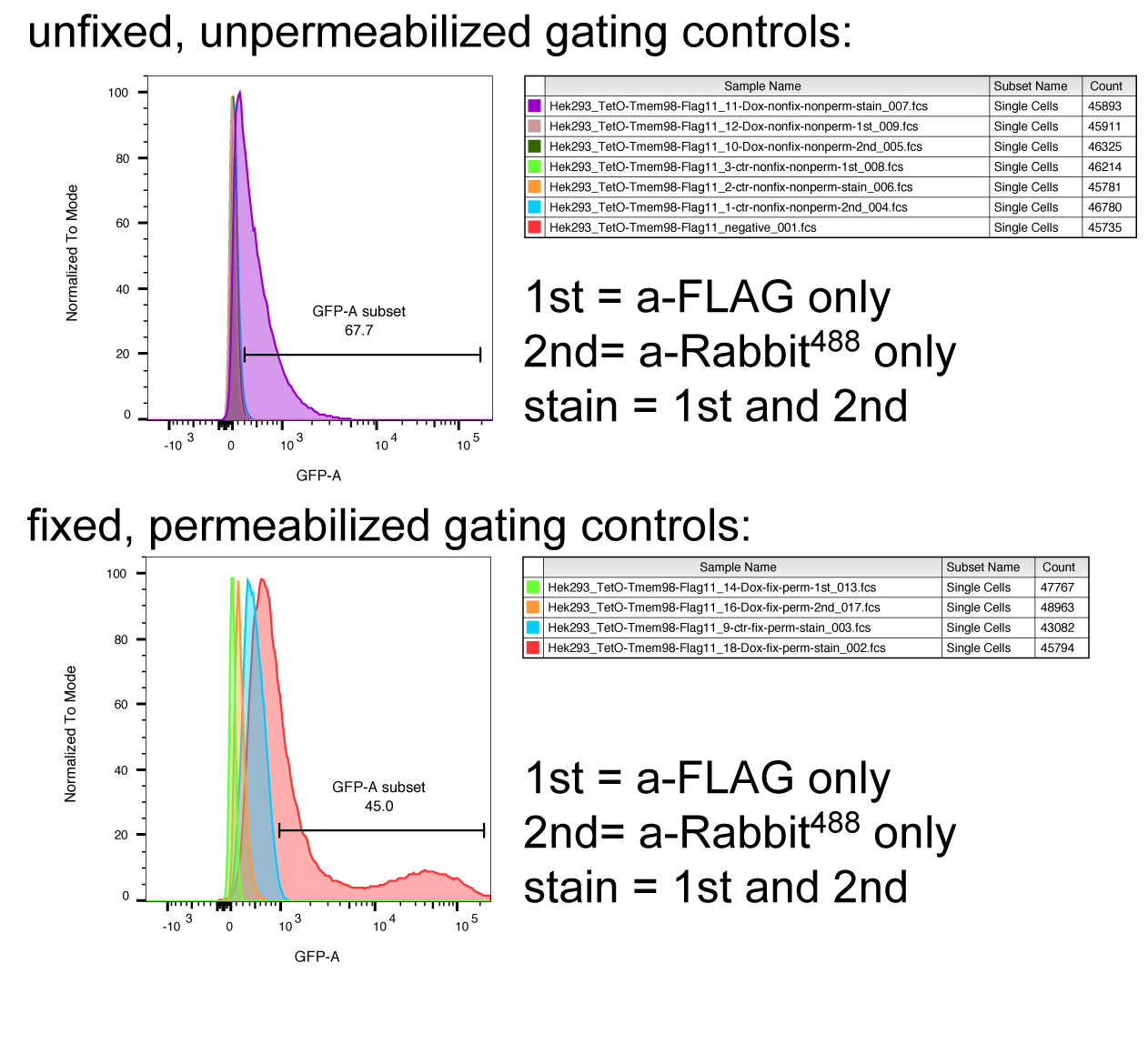

Supplement: S4 Fig — (TIF) [file pone.0227435.s004.tif]

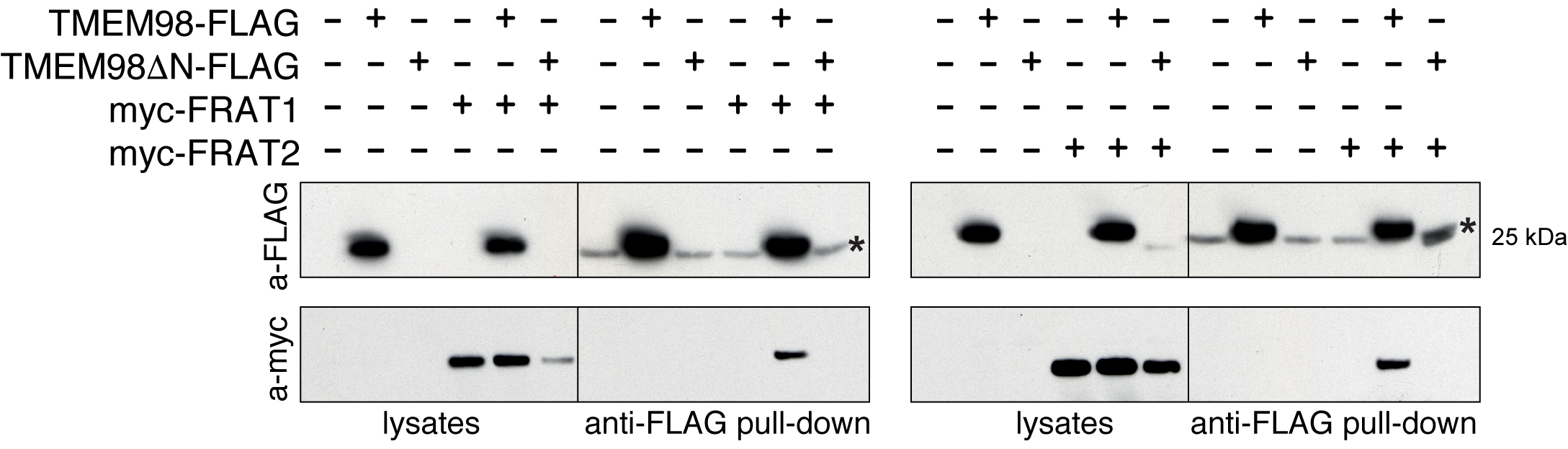

Supplement: S5 Fig — (TIF) [file pone.0227435.s005.tif]

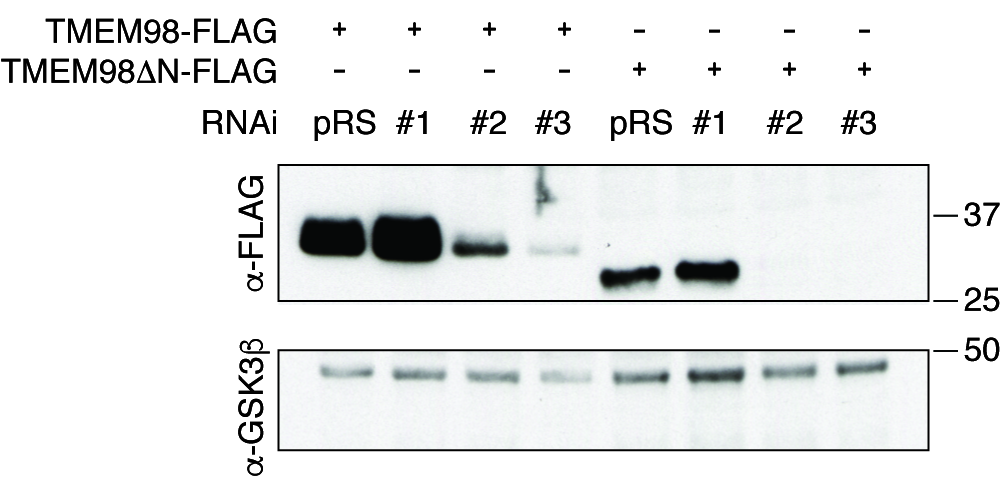

Supplement: S6 Fig — (TIF) [file pone.0227435.s006.tif]

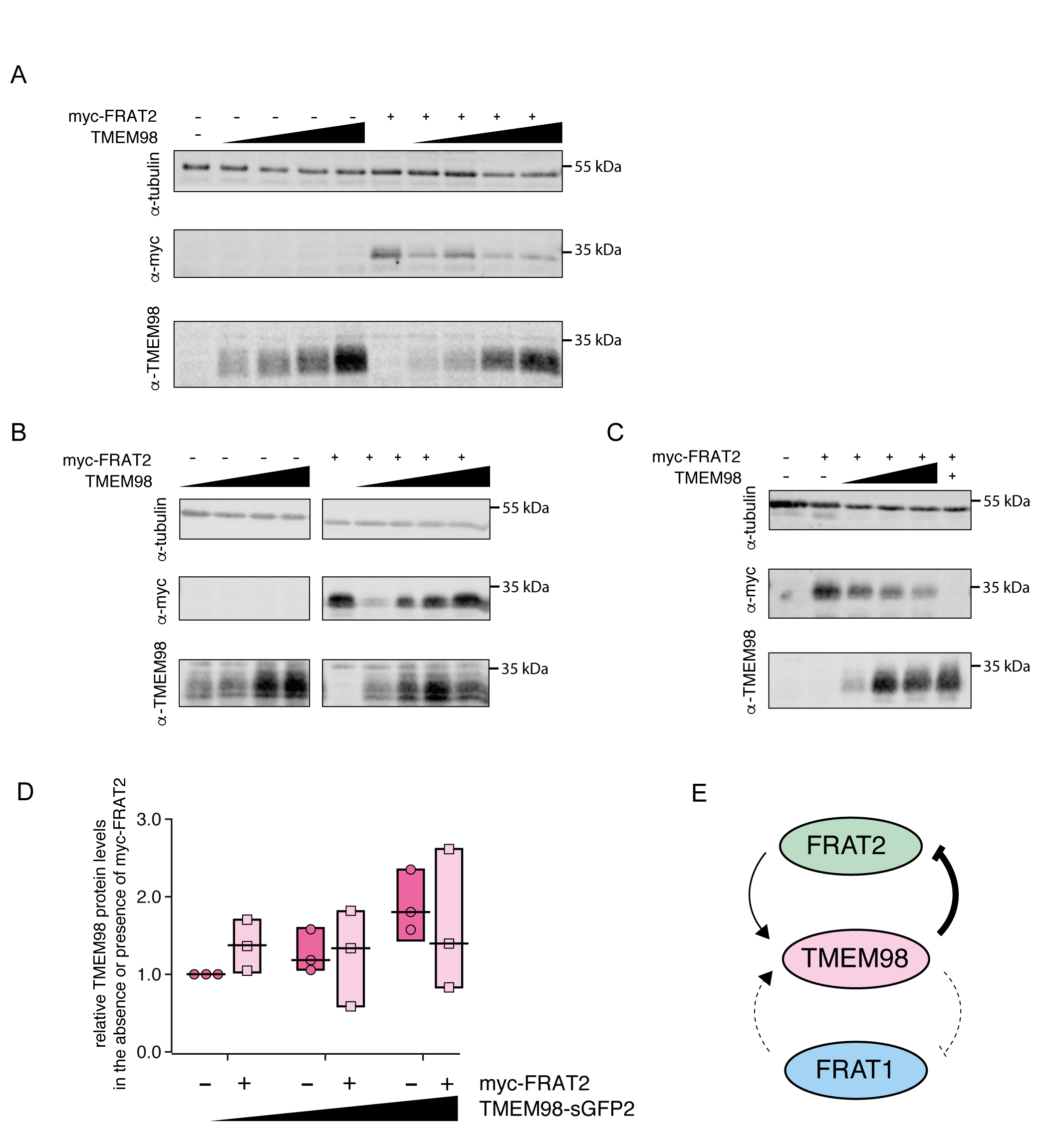

Supplement: S7 Fig — (TIF) [file pone.0227435.s007.tif]

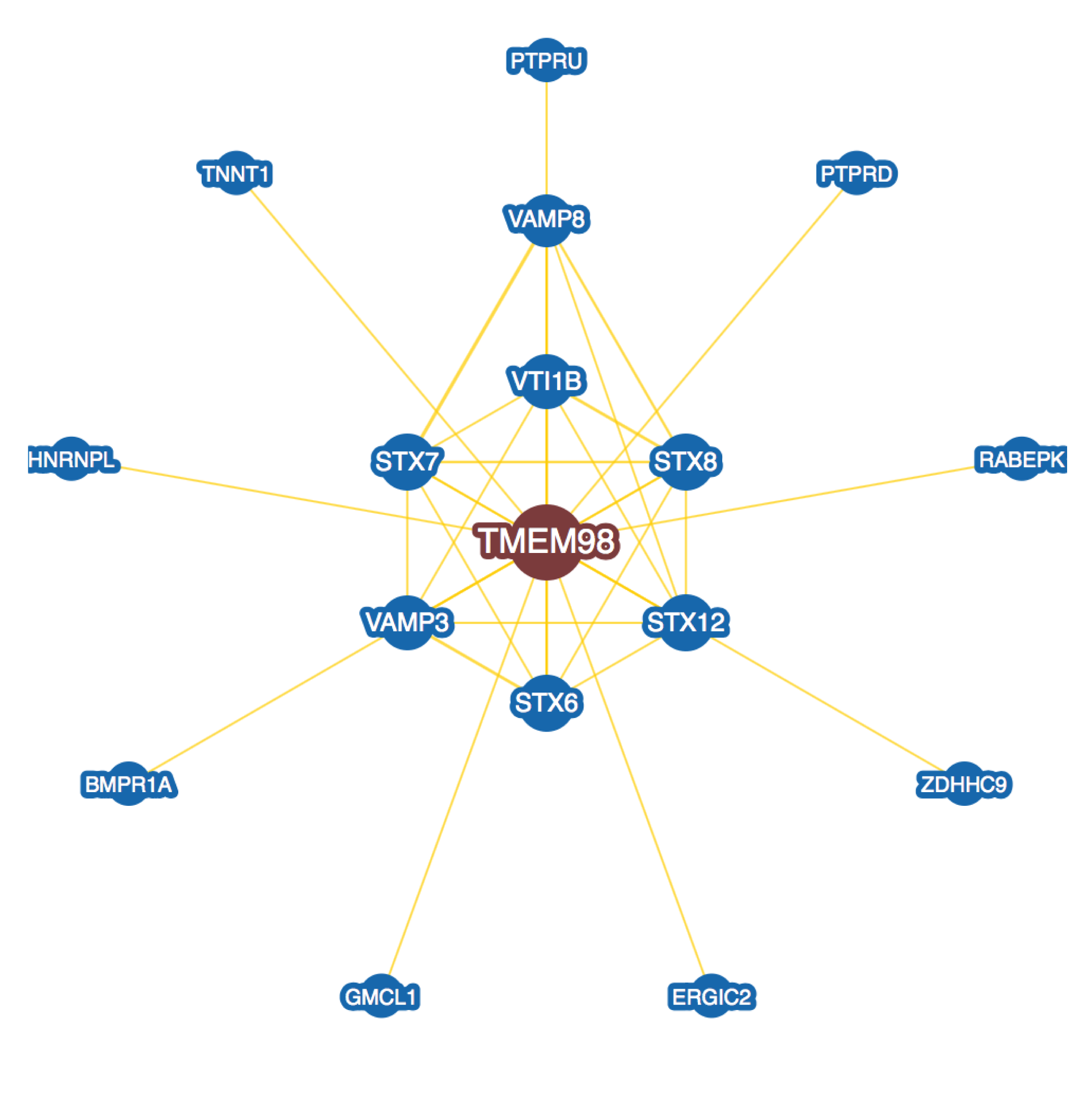

Supplement: S8 Fig — (TIF) [file pone.0227435.s008.tif]

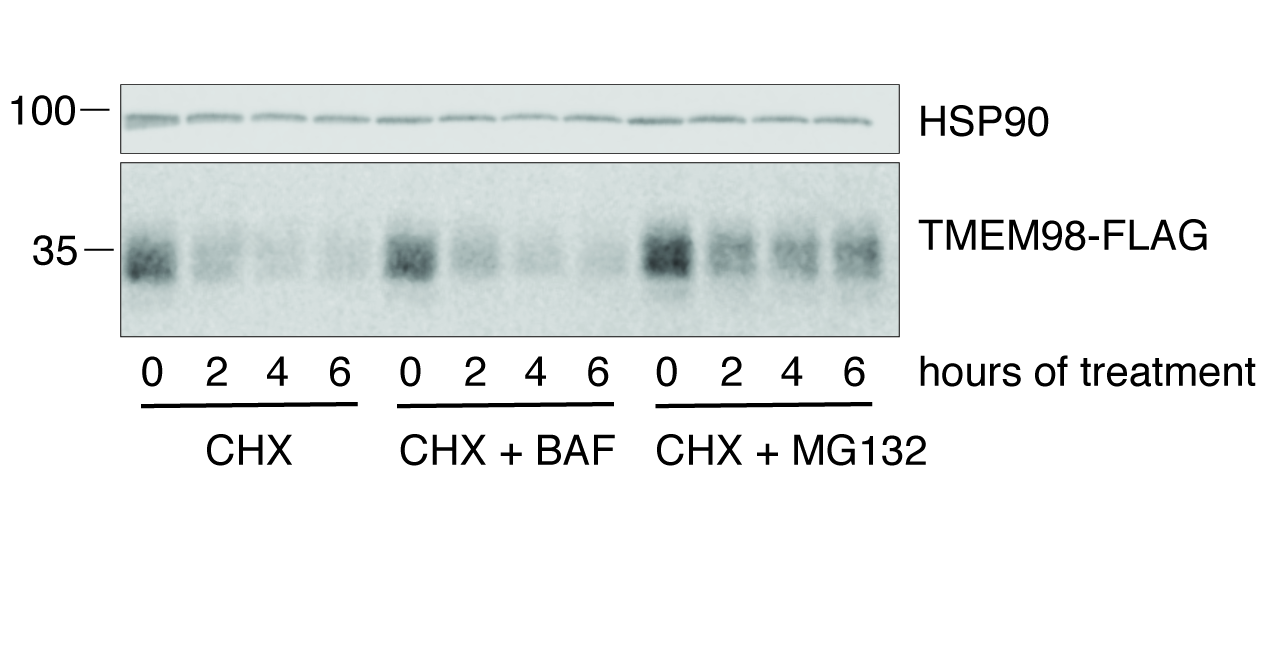

Supplement: S9 Fig — (TIF) [file pone.0227435.s009.tif]
